# Fenótipo Hipertrófico em Adolescente da Região Amazônica com Variantes Combinadas nos genes GLA, TTR e PRKAG2

**DOI:** 10.36660/abc.20260122

**Published:** 2026-07-31

**Authors:** Matheus Martins Monteiro, Kemelly Ferreira da Silva, Jessica Vanina Ortiz, Marcos Leite Barbosa Ferreira, Kátia do Nascimento Couceiro, Rodrigo Fernandes de Castro, Fábio Fernandes, João Marcos Bemfica Barbosa Ferreira, Investigadores do Mapa Genoma Brasil, Bianca Domit Wener Linnenkamp, Emanuelle Leonilia Marques, José Eduardo Krieger

**Affiliations:** 1 A Real e Benemérita Associação Portuguesa de Beneficência São Paulo SP Brasil A Real e Benemérita Associação Portuguesa de Beneficência, São Paulo, SP – Brasil; 2 Universidade Nilton Lins Manaus AM Brasil Universidade Nilton Lins, Manaus, AM – Brasil; 3 Universidade do Estado do Amazonas Manaus AM Brasil Universidade do Estado do Amazonas, Manaus, AM – Brasil; 4 Hospital das Clínicas Faculdade de Medicina Universidade de São Paulo São Paulo SP Brasil Instituto do Coração do Hospital das Clínicas da Faculdade de Medicina da Universidade de São Paulo, São Paulo, SP – Brasil

**Keywords:** Amiloidose Familiar, Cardiomiopatia Hipertrófica, Doença de Fabry

## Introdução

A cardiomiopatia hipertrófica (CMH) é definida como um aumento da espessura da parede do ventrículo esquerdo e/ou direito sem condições de sobrecarga que expliquem esse aumento. O diagnóstico é confirmado por exames de imagem, como ecocardiograma e ressonância magnética cardíaca (RMC), com a presença de espessura da parede ≥ 15 mm em qualquer segmento miocárdico ou ≥ 13 mm em parentes de primeiro grau de pacientes com diagnóstico confirmado.^1^ A causa mais comum desse fenótipo é a CMH genética, causada por mutações em genes que codificam proteínas sarcoméricas. A maioria dos pacientes com CMH é assintomática, mas alguns podem apresentar sintomas significativos, sendo essa uma importante causa de morte súbita em jovens.^2^ No entanto, é importante considerar outras condições conhecidas como fenocópias, pois podem apresentar características semelhantes em exames de imagem, mas com etiologias específicas e mecanismos fisiopatológicos distintos. Entre essas fenocópias, destacam-se as doenças mitocondriais, as RASopatias, as distrofias musculares, a síndrome PRKAG2, a doença de Fabry e a amiloidose cardíaca. O diagnóstico diferencial é essencial, pois essas fenocópias apresentam estratégias diagnósticas e terapêuticas específicas que podem impactar a evolução natural da doença.^3^ O presente relato descreve o caso de um paciente jovem em que esse diagnóstico diferencial foi desafiador, tanto pelas dificuldades de acesso a exames específicos quanto pela sobreposição de doenças que dificultaram o diagnóstico, e descreve a distribuição da doença em sua família. Este paciente faz parte de um projeto de pesquisa mais amplo, aprovado pelo Comitê de Ética em Pesquisa da Universidade do Estado do Amazonas sob os números de aprovação 7.108.530 e CAAE 55200322.5.3009.516.

## Relato de Caso

Paciente do sexo masculino, nascido e residente em Manaus, Amazonas, Brasil, que, aos 12 anos de idade, recebeu o diagnóstico de CMH obstrutiva após internação por meningite bacteriana. Na ocasião, seu ecocardiograma mostrou diâmetro diastólico do ventrículo esquerdo (DDVE) de 33 mm, espessura do septo interventricular de 25 mm e espessura da parede posterior de 27 mm, com fração de ejeção do ventrículo esquerdo (FEVE) de 88% e presença de gradiente de 38 mmHg na via de saída do ventrículo esquerdo (VSVE) em repouso. Em seu histórico familiar, ambos os pais e dois irmãos estavam vivos e sem sintomas cardiovasculares. Permaneceu assintomático até os 16 anos, quando procurou atendimento no serviço de cardiologia com episódios de palpitações, dispneia e dor torácica. O exame físico não revelou anormalidades neurológicas ou dermatológicas. A ausculta cardíaca demonstrou sopro sistólico na borda esternal esquerda, no quarto espaço intercostal. O eletrocardiograma revelou o eixo QRS no quadrante superior direito, presença de ondas Q na parede lateral, sobrecarga biventricular e alteração da repolarização ventricular de V1 a V6 (Figura 1). Um novo ecocardiograma transtorácico bidimensional mostrou um DDVE de 40 mm, espessura do septo interventricular de 52 mm e espessura da parede posterior de 40 mm, com FEVE de 63% e gradiente da VSVE de 141 mmHg em repouso (Figura 2). Foi realizado um Holter de 24 horas, que mostrou um episódio de taquicardia ventricular não sustentada. Os exames de função renal, como ureia, creatinina e uranálise, estavam dentro dos limites normais. A RMC e a cintilografia cardíaca com radiotraçadores osteotrópicos não foram realizadas devido a questões logísticas. Ele iniciou o tratamento com succinato de metoprolol 50 mg duas vezes ao dia e foi solicitada a implantação de um cardioversor-desfibrilador implantável (CDI). A possibilidade de transplante cardíaco foi discutida; no entanto, esse procedimento não estava disponível na cidade do paciente. Iniciou-se então um processo de tratamento fora de casa. Nesse momento, foi realizado o teste genético. Ele retornou para acompanhamento um mês depois, apresentando alívio parcial dos sintomas e redução do gradiente da VSVE para 53 mmHg. No entanto, enquanto aguardava a implantação do CDI e a validação para o transplante cardíaco, ele faleceu subitamente em casa.

**Figura 1:**
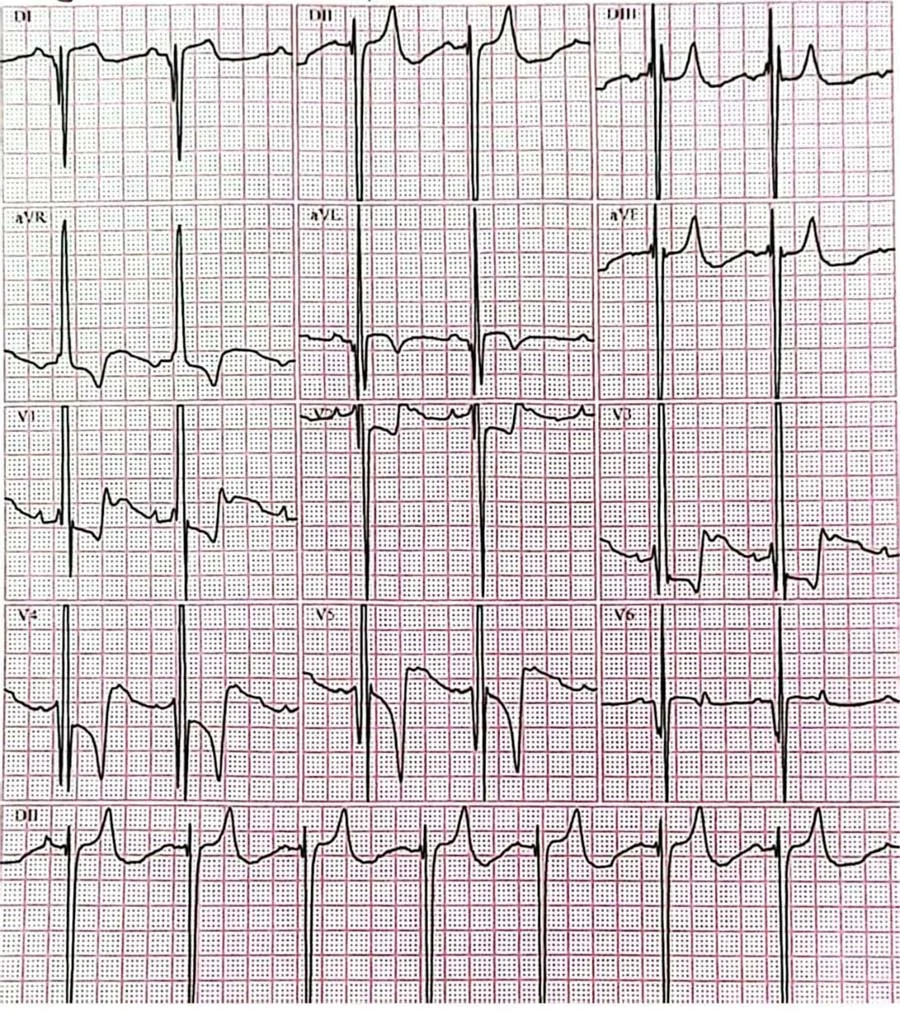
– ECG mostrando desvio extremo do eixo, presença de ondas Q na parede lateral, sobrecarga biventricular e repolarização ventricular alterada de V1 a V6.

**Figura 2:**
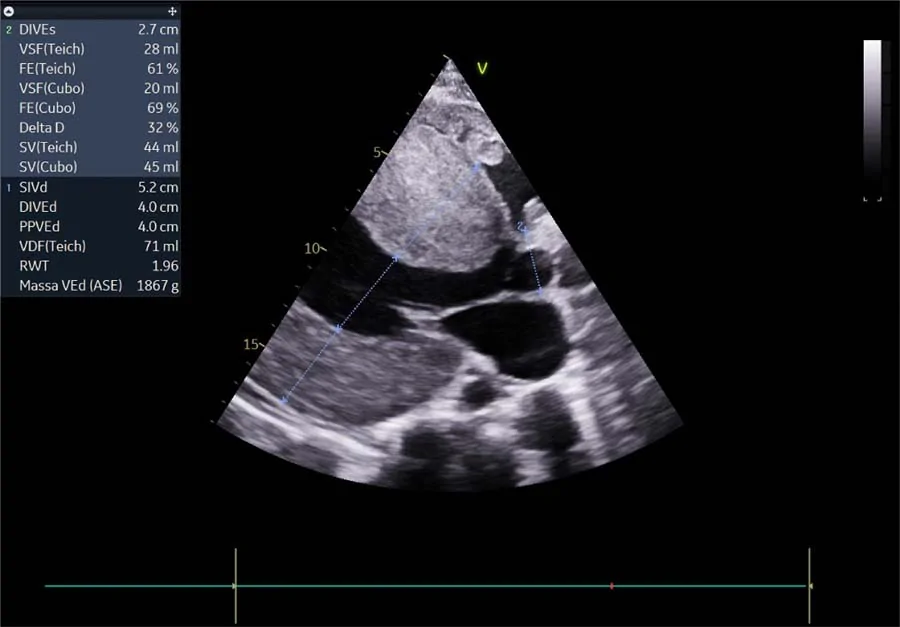
– Ecocardiograma transtorácico bidimensional mostrando uma espessura do septo interventricular de 52 mm, uma espessura da parede posterior de 40 mm e FEVE de 63%.

O teste genético mostrou a presença de três variantes simultaneamente: (1) uma variante patogênica em hemizigose no gene GLA, descrita como NM_000169.3(GLA):c.870G>A;p.(Met290Ile) associada à doença de Fabry; (2) uma variante provavelmente patogênica no gene TTR heterozigoto descrita como NM_000371.4(TTR):c.289T>C;p.(Ser97Pro), associada à amiloidose hereditária relacionada à transtirretina e (3) uma variante de significado incerto no gene PRKAG2 heterozigoto, descrita como NM_016203.4(PRKAG2):c.969C>A;p.(Phe323Leu). Na investigação da doença de Fabry, a atividade da alfa-galactosidase (GLA) apresentou alteração com uma dosagem de 0,66 mcmol/L/h (VR: >1,68 mcmol/L/h). A dosagem do biomarcador Lyso-Gb 3 foi de 0,85 ng/mL (VR: 1,1 ng/mL).

Os testes em parentes de primeiro grau revelaram a ausência de variantes no pai, a presença da mesma variante patogênica em hemizigose no gene GLA no irmão mais velho e a presença das mesmas variantes nos genes GLA e TTR na mãe e na irmã mais nova. Os testes em parentes de primeiro grau revelaram a ausência de variantes no pai, a presença da mesma variante patogênica em hemizigose no gene GLA no irmão (21 anos) e a presença das mesmas variantes nos genes GLA e TTR na mãe (40 anos) e na irmã (14 anos). No irmão, a atividade da alfa-galactosidase A (GLA) foi de 0,75 µmol/L/h (VR: ≥ 1,68 µmol/L/h) e a dosagem do biomarcador Lyso-Gb 3 foi de 1 ng/mL (VR: 1,1 ng/mL). Na mãe, a atividade da alfa-galactosidase A (GLA) foi de 1,82 µmol/L/h (VR: ≥ 1,68 µmol/L/h) e a dosagem do biomarcador Lyso-Gb 3 foi de 0,4 ng/mL (VR: 1,1 ng/mL). Na irmã, a atividade da alfa-galactosidase A (GLA) foi de 3,48 µmol/L/h (VR: ≥ 1,68 µmol/L/h) e a dosagem do biomarcador Lyso-Gb 3 foi de 0,4 ng/mL (VR: 1,1 ng/mL). Os demais membros da família permanecem assintomáticos. O heredograma é apresentado na Figura 3.

**Figura 3:**
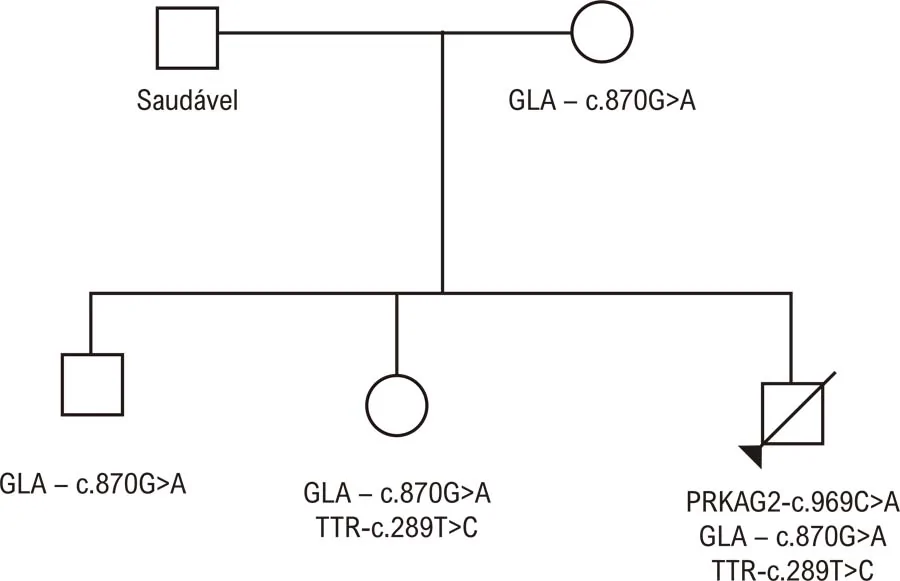
– Árvore genealógica da família.

## Discussão

O presente relato descreve uma rara combinação de três variantes genéticas simultâneas, GLA, TTR e PRKAG2, resultando em um fenótipo complexo de CMH, sendo difícil definir qual variante causa a doença ou se houve um efeito sinérgico. O uso da RMC para avaliar o padrão de realce miocárdico tardio poderia auxiliar no diagnóstico. Além disso, a cintilografia miocárdica com pirofosfato poderia sugerir amiloidose cardíaca, caso presente. Ao mesmo tempo, uma biópsia miocárdica poderia confirmar o diagnóstico. Infelizmente, esses exames não estão disponíveis em uma região com acesso limitado a exames mais complexos. Portanto, podemos inferir o efeito potencial de cada variante a partir da epidemiologia específica de cada doença.

O gene PRKAG2 pode estar relacionado ao processo de transcrição da proteína quinase ativada por AMP (AMPK), sendo sua mutação responsável pela perda de função da subunidade γ2 da proteína ou por um defeito no processo de glicogênio. Pacientes com mutações nesse gene apresentam principalmente aumento da espessura ventricular associado a distúrbios da condução cardíaca, como na síndrome de pré-excitação ventricular. Essa alteração genética é considerada rara, estando mais associada ao início precoce e à evolução de alterações cardíacas em pacientes mais jovens.^4,5^

Outra cardiomiopatia com fenótipo hipertrófico considerada rara é a doença de Fabry. Esta doença é caracterizada pela formação de armadura lisossomal, com deposição progressiva de glicoesfingolipídios (GL-3) nos tecidos, podendo comprometer diversos sistemas, incluindo o cardiovascular. É causada por uma mutação no gene GLA com herança ligada ao cromossomo X. Dois fenótipos são descritos na doença de Fabry: o fenótipo clássico, com envolvimento grave, precoce e multissistêmico, principalmente em homens, e o fenótipo tardio, que pode ocorrer em homens ou mulheres e geralmente apresenta envolvimento cardíaco isolado.^6,7^

Dentre as mutações genéticas responsáveis pelas cardiomiopatias hipertróficas, destaca-se a amiloidose, que causa deposição de proteínas fibrilares em órgãos como coração, rins e sistema nervoso. A forma cardíaca apresenta aumento da espessura ventricular, combinado com aumento da rigidez das câmaras. A amiloidose cardíaca, devido à deposição de transtirretina (ATTR), pode ser frequentemente causada por variantes genéticas no gene TTR e, portanto, afeta pacientes mais idosos.

A idade de 16 anos é um fator determinante neste caso, visto que tanto a doença de Fabry, em sua forma fenotípica tardia, quanto a amiloidose causada pela transtirretina geralmente se manifestam em idades mais avançadas. Da mesma forma, a doença de Fabry com características fenotípicas clássicas apresenta uma abordagem multissistêmica, o que não ocorreu neste caso.^8-10^

A síndrome PRKAG2 afeta jovens entre 10 e 20 anos de idade e está fortemente associada a taquiarritmias ventriculares malignas e bloqueios atrioventriculares graves. Portanto, essa mutação pode ser o principal determinante dessa doença fatal precoce. Possivelmente, variantes nos genes TTR e GLA são apenas fatores secundários.

## Conclusão

Este relato de caso, envolvendo três variantes genéticas associadas ao aumento da espessura da parede do ventrículo esquerdo, enfatiza a relevância do diagnóstico diferencial em doenças de depósito cardíaco. A determinação precisa da causa subjacente é essencial para orientar intervenções específicas e prevenir complicações irreversíveis. Métodos diagnósticos avançados, incluindo RMC com mapeamento T1, testes genéticos e biópsia endomiocárdica, são essenciais para a caracterização dessas condições, permitindo a individualização do tratamento e um melhor prognóstico para os pacientes. Sabe-se que a síndrome PRKAG2 afeta jovens entre 10 e 20 anos de idade. Assim, neste caso, hipotetizamos que essa mutação poderia ser o principal determinante dessa doença precoce e fatal. No entanto, acreditamos que testes mais específicos, como RMC e biópsia endomiocárdica, seriam fundamentais para a confirmação diagnóstica. Este caso nos leva a refletir sobre a dificuldade diagnóstica do fenótipo de CMH.
