# Supplementary material for: Fenótipo Hipertrófico em Adolescente da Região Amazônica com Variantes Combinadas nos genes GLA, TTR e PRKAG2
Source: Arq Bras Cardiol. 2026 Jul 31;123(7):e20260122. [Article in Portuguese] doi: 10.36660/abc.20260122 (PMC13718332; doi:10.36660/abc.20260122)
Supplement: Translation

# Translation: Hypertrophic Phenotype in an Adolescent from the Amazon Region Associated with Combined Variants in the GLA, TTR, and PRKAG2 genes

**DOI:** 10.36660/abc.20260122i

**Authors:** Matheus Martins Monteiro, Kemelly Ferreira da Silva, Jessica Vanina Ortiz, Marcos Leite Barbosa Ferreira, Kátia do Nascimento Couceiro, Rodrigo Fernandes de Castro, Fábio Fernandes, João Marcos Bemfica Barbosa Ferreira, Investigadores do Mapa Genoma Brasil, Bianca Domit Wener Linnenkamp, Emanuelle Leonilia Marques, José Eduardo Krieger

**Affiliations:** 1 Brazil A Real e Benemérita Associação Portuguesa de Beneficência, São Paulo, SP – Brazil; 2 Brazil Universidade Nilton Lins, Manaus, AM – Brazil; 3 Brazil Universidade do Estado do Amazonas, Manaus, AM – Brazil; 4 Brazil Instituto do Coração do Hospital das Clínicas da Faculdade de Medicina da Universidade de São Paulo, São Paulo, SP – Brazil

**Keywords:** Familial Amyloidosis, Hypertrophic Cardiomyopathy, Fabry Disease

## Introduction

Hypertrophic cardiomyopathy (HCM) phenotype is defined as increased wall thickness of the left and/or right ventricle without loading conditions that explain this. Its diagnosis is confirmed by imaging tests such as echocardiogram and cardiac magnetic resonance (CMR) with the presence of wall thickness ≥ 15 mm in any myocardial segment or 13 mm in first-grade relatives of patients with a confirmed diagnosis.^1^ The most common cause of this phenotype is genetic HCM caused by mutations in genes encoding sarcomeric proteins. Most patients with HCM are asymptomatic, but some patients may present significant symptoms, and this is an important cause of sudden death in young people.^2^ However, it is important to consider other conditions known as phenocopies because they may have similar characteristics in imaging tests, but with specific etiologies that have different pathophysiological mechanisms. Among these phenocopies, we highlight mitochondrial diseases, RASopathies, muscular dystrophies, PRKAG2 syndrome, Fabry disease, and cardiac amyloidosis. Their differential diagnosis is essential because these phenocopies present specific diagnostic and therapeutic strategies that can impact the natural evolution of disease.^3^ The present report reveals the case of a young patient in which this differential diagnosis was challenging, both due to difficulties in accessing specific exams and the overlapping of diseases that made his diagnosis hard, and describes the disease distribution in his family. This patient is part of a broader research project approved by the Research Ethics Committee of the Universidade do Estado do Amazonas under approval number 7.108.530 and CAAE 55200322.5.3009.516.

## Case Report

Male patient, born and resident in Manaus, Amazonas, Brazil, who, at 12 years old, received a diagnosis of obstructive HCM after being hospitalized for bacterial meningitis. At that time, his echocardiogram showed left ventricular diastolic diameter (LVDD) of 33 mm, interventricular septum thickness of 25 mm, and posterior wall thickness of 27 mm, with left ventricular ejection fraction (LVEF) of 88% and presence of left ventricular outflow tract (LVOT) gradient of 38 mmHg at rest. In his family history, he had both parents and two siblings alive and without any cardiovascular symptoms. He remained asymptomatic until the age of 16, when he sought care in the cardiology service with episodes of palpitations, dyspnea, and chest pain. The physical examination did not reveal any neurological or dermatological abnormalities. Cardiac auscultation demonstrated a systolic murmur on the left sternal border, fourth intercostal space. The electrocardiogram revealed the QRS axis in the right upper quadrant, presence of Q waves in the lateral wall, biventricular overload, and altered ventricular repolarization from V1 to V6. (Figure 1). A new two-dimensional transthoracic echocardiogram showed a LVDD of 40 mm, an interventricular septum thickness of 52 mm, and a posterior wall thickness of 40 mm, with LVEF of 63% and LVOT gradient of 141 mmHg at rest (Figure 2). A 24-hour Holter was performed, which showed an episode of non-sustained ventricular tachycardia (NSVT). Kidney function tests, such as urea, creatinine, and urinalysis, were within normal limits. CMR imaging and cardiac scintigraphy with bone-seeking radiotracers were not performed due to logistical issues. He started treatment with metoprolol succinate 50 mg twice a day, and implantation of an implantable cardioverter-defibrillator (ICD) was requested. The possibility of a heart transplant was discussed; however, this procedure was not available in the patient’s city. A process for treatment outside the home was initiated. At that time, genetic testing was conducted. He returned for follow-up one month later with partial relief of symptoms and a reduction of LVOT gradient to 53 mmHg. However, while awaiting the implantation of the ICD and approval for a heart transplant, he passed away suddenly at home.

**Figure 1:** – ECG showing extreme axis deviation, presence of Q waves in the lateral wall, biventricular overload, and altered ventricular repolarization from V1 to V6.

**Figure 2:** – Two-dimensional transthoracic echocardiogram showing an interventricular septum thickness of 52 mm, a posterior wall thickness of 40 mm, and LVEF of 63%.

The genetic testing showed the presence of three variants simultaneously: (1) a pathogenic variant in hemizygosity in the GLA gene, described as NM_000169.3(GLA):c.870G>A;p.(Met290Ile) associated with Fabry disease; (2) a probably pathogenic variant in the heterozygous TTR gene described as NM_000371.4(TTR):c.289T>C;p.(Ser97Pro), associated with hereditary amyloidosis related to transthyretin and (3) a variant of uncertain significance in the heterozygous gene PRKAG2, described as NM_016203.4(PRKAG2):c.969C>A;p.(Phe323Leu). In the investigation of Fabry disease, the activity of alpha-galactosidase (GLA) showed alteration with a dosage of 0.66 mcmol/L/h (VR: >1.68 mcmol/L/h). The Lyso-Gb 3 biomarker dosage was 0.85 ng/mL (VR: 1.1 ng/mL).

Testing of first-degree relatives revealed the absence of variants in the father, the presence of the same pathogenic variant in hemizygosity in the GLA gene in the older sibling (male), and the presence of the same variants in the GLA and TTR genes in the mother and the youngest sibling (female). Testing of first-degree relatives revealed the absence of variants in the father, the presence of the same pathogenic variant in hemizygosity in the GLA gene in the brother (21 years old), and the presence of the same variants in the GLA and TTR genes in the mother (40 years old) and the sister (14 years old). In the brother, the activity of alpha-galactosidase A (GLA) was 0.75 mcmol/L/h (VR: ≥ 1.68 mcmol/L/h), and the dosage of the Lyso-Gb 3 biomarker was 1 ng/mL (VR: 1.1 ng/mL). In the mother, the activity of alpha-galactosidase A (GLA) was 1.82 mcmol/L/h (VR: ≥ 1.68 mcmol/L/h), and the dosage of biomarker Lyso-Gb 3 was 0.4 ng/mL (VR: 1.1 ng/mL). In the sister, the activity of alpha-galactosidase A (GLA) was 3.48 mcmol/L/h (VR: ≥ 1.68 mcmol/L/h), and the dosage of the Lyso-Gb 3 biomarker was 0.4 ng/mL (VR: 1.1 ng/mL). The family members remain asymptomatic. The family members remain asymptomatic. The heredogram is shown in Figure 3.

**Figure 3:** – Family pedigree.

## Discussion

The present report described a rare combination of three simultaneous genetic variants GLA, TTR, and PRKAG2 resulting in a complex phenotype of HCM, which is difficult to define which variant causes the disease or if there was a synergistic effect. The use of CMR to evaluate the pattern of late myocardial enhancement could assist in diagnosis. Furthermore, performing myocardial scintigraphy with pyrophosphate could suggest cardiac amyloidosis if present. At the same time, a myocardial biopsy could define the diagnosis. Unfortunately, these exams are not available in a region with difficult access to more complex exams. Therefore, we can infer the potential effect of each variant from the epidemiology of each disease specifically.

PRKAG2 is a gene that may be related to the transcription process of AMP-activated protein kinase (AMPK), with its mutation responsible for the loss of function of the y2 subunit of the protein, or that results in a defect in the glycogen process. Patients with gene mutations evolve mainly because of increased ventricular thickness associated with abnormal cardiac conduction, such as the case of ventricular pre-excitation syndrome. This genetic alteration is considered rare, being more associated with the early onset and evolution of cardiac alterations in younger patients.^4,5^

Another cardiomyopathy with a hypertrophic phenotype considered rare is Fabry disease. This disease is characterized by lysosomal armature, with progressive deposition of glyconesphingolipids (GL-3) in tissues, potentially compromising several systems, including the cardiovascular system. It is caused by a mutation in the GLA gene with X-linked inheritance. Two phenotypes are described in the Fabry disease: the classic phenotype with severe, early, and multisystem involvement, mainly in men, and the late phenotype that can occur in men or women and generally has an isolated cardiac involvement.^6,7^

Among the genetic mutations responsible for hypertrophic cardiomyopathies, amyloidosis also stands out, which causes deposition of fibrillar proteins in organs such as the heart, kidneys, and nervous system. The cardiac form presents an increase in ventricular thickness, combined with an increase in chamber rigidity. Cardiac amyloidosis, due to deposition of transthyretin (ATTR), can frequently be caused by genetic variants in the TTR gene and therefore affects older pacients.

The age of 16 is a determining factor in this case, as both the Fabry disease, in its late phenotypic form, and the amyloidosis caused by TTR generally manifest themselves in more advanced ages. Likewise, Fabry disease with classical phenotypic features presents with a multisystem approach, which did not happen in this case.^8-10^

The PRKAG2 syndrome is known to affect young people between 10 and 20 years of age and is strongly associated with malignant ventricular tachyarrhythmias and severe atrioventricular blocks. Thus, this mutation could be the main determinant of this early fatal disease. Possibly, variants in the TTR and GLA genes are most likely bystanders.

## Conclusion

This case report, involving three genetic variants associated with increased left ventricular wall thickness, emphasizes the relevance of differential diagnosis in cardiac deposition disorders. A precise determination of the underlying cause is essential to guide specific interventions and prevent irreversible complications. Advanced diagnostic methods, including magnetic resonance imaging with T1 mapping, genetic tests, and endomyocardial biopsy, are essential for the characterization of these conditions, allowing individualization of treatment and better prognosis for patients. PRKAG2 syndrome is known to affect young people between 10 and 20 years of age. Thus, in this case, we hypothesized that this mutation could be the main determinant of this early fatal disease. However, we believe that more specific tests, such as CMR and endomyocardial biopsy, would be fundamental for diagnostic confirmation. This case brings us to reflect on the difficult diagnosis of the HCM phenotype.
